# Advances in Molecular Genetics and Breeding of Cattle, Sheep, and Goats

**DOI:** 10.3390/ani16081130

**Published:** 2026-04-08

**Authors:** Xiukai Cao

**Affiliations:** Joint International Research Laboratory of Agriculture and Agri-Product Safety of Ministry of Education of China, Yangzhou University, Yangzhou 225009, China; cxkai0909@163.com

## 1. Introduction

Ruminant livestock—cattle, sheep, and goats—are cornerstones of global food security, collectively providing meat, milk, fiber, and other essential products that sustain the livelihoods of hundreds of millions of people across diverse agro-ecological zones [1]. As the global human population is expected to approach ten billion, the livestock sector faces an intensifying dual challenge: enhancing productivity and product quality while simultaneously improving resilience to climate change, emerging infectious diseases, and the societal imperative to reduce the environmental footprint of animal agriculture. Traditional breeding programs, which rely on phenotypic selection and pedigree information, have delivered steady but incremental genetic gains; however, they remain constrained by long generation intervals, the high cost of progeny testing, and limited ability to dissect the genetic architecture of complex, polygenic traits.

The genomic era has fundamentally transformed this landscape. The construction of reference genomes for cattle [2], sheep [3], and goats [4] has provided the essential framework for high-resolution genetic analyses, while the subsequent development of high-density single nucleotide polymorphism (SNP) arrays and whole-genome resequencing platforms has enabled genome-wide association studies (GWAS) and genomic selection (GS) at population scale. The landmark proposal by Meuwissen et al. of genomic selection using genome-wide dense marker maps [5] catalyzed a paradigm shift in dairy cattle breeding, which is now extending to sheep, goat, and beef cattle systems [6]. Concurrently, multi-omics technologies—integrating transcriptomics, epigenomics, proteomics, and metabolomics—have opened new windows into the molecular mechanisms connecting genotype to phenotype, highlighting the contributions of non-coding RNAs, epigenetic modifications, and post-translational regulation in shaping complex traits [7].

Genome-editing technologies, particularly CRISPR-Cas systems [8,9], have further expanded the toolkit of animal breeders, enabling the precise introduction or disruption of targeted alleles and offering the prospect of accelerating genetic improvement beyond what conventional selection can achieve. Alongside these biotechnological advances, the adoption of machine learning algorithms for genomic prediction has attracted growing attention, with ensemble methods demonstrating the potential to model non-linear marker effects and improve prediction accuracy for traits of economic importance [10].

Against this backdrop, the Topic “Advances in Molecular Genetics and Breeding of Cattle, Sheep, and Goats” was launched to consolidate cutting-edge research across these intersecting disciplines. The Topic attracted 35 contributions—33 original research articles and two comprehensive reviews—spanning genomic selection, functional gene characterization, non-coding RNA biology, multi-omics analyses, molecular mechanisms of economic traits, adaptive traits, and disease resistance. This editorial provides a thematic overview of these contributions and highlights their collective significance for advancing precision breeding and sustainable ruminant production.

## 2. An Overview of Published Articles

### 2.1. Genetic Adaptation to Extreme Environments

Several contributions examined how ruminants have evolved molecular strategies to cope with high-altitude hypoxia, cold stress, and other environmental extremes—challenges of particular relevance to plateau and mountain regions where indigenous breeds must thrive under conditions that impose strong natural selection.

A study on indigenous Yunnan goats distributed along an altitudinal gradient (500–3500 m) identified a non-synonymous SNP (g.86650A>T, p.Gln556Leu) in the *EPAS1* gene—a master regulator of the hypoxia-inducible factor (HIF) pathway—whose frequency increased significantly with elevation. The T allele was associated with elevated red blood cell counts and hemoglobin concentrations, while simultaneously reducing erythrocyte aggregation to maintain blood flow efficiency. Protein structural modeling confirmed the variant’s functional significance, providing a molecular basis for high-altitude adaptation in goats analogous to mechanisms documented in Tibetan yak and high-altitude human populations [11]. In yak placenta, the adhesion molecules *CTNNB1* and *CDH1* were shown to regulate trophoblast cell adhesion and tight junction formation across gestational stages under hypoxic conditions, illuminating the maternal-fetal molecular interface essential for successful reproduction at altitude. Cold-stress resilience was addressed in bovine mammary epithelial cells, where ENO1—a glycolytic enzyme—was identified as a critical modulator of apoptosis and energy metabolism under acute cold exposure, offering a candidate marker for maintaining lactation performance in dairy systems subject to seasonal temperature extremes.

### 2.2. Wool, Fiber, and Coat Quality Genetics

Fiber quality is a paramount economic trait for the sheep and cashmere goat industries, and a substantial cluster of articles advanced the molecular genetics of wool and cashmere fiber production.

The keratin-associated protein (*KRTAP*) gene family, which encodes the matrix proteins that determine fiber ultrastructure and mechanical properties, was examined in multiple contexts. Polymorphisms in *KRTAP13-3* were associated with wool diameter, heterotypic fiber variability, and uniformity in Chinese Tan sheep. A newly identified gene, *KRTAP6-3*, was characterized for the first time in *Capra hircus*; its genetic analysis revealed significant associations with cashmere fiber diameter, identifying a new target for genetic improvement in the luxury fiber market. The *KRT71* keratin gene was examined in Gansu Alpine Fine-Wool Sheep, with KASP genotyping and immunofluorescence demonstrating that its polymorphisms influence wool length and that its expression is localized to the inner root sheath of the hair follicle.

A comprehensive review synthesized current knowledge of wool fiber properties, structure, and the genetic strategies available for sheep improvement, serving as an authoritative reference bridging molecular biology and field-applicable breeding. Complementing the keratin-gene studies, a screening study identified circRNAs associated with secondary wool follicle (SF) development in fine-wool sheep and constructed a competing endogenous RNA (ceRNA) network involving the Wnt and TGF-β pathways, establishing that non-coding RNA-mediated post-transcriptional regulation is an important dimension of SF morphogenesis. The genetics of coat color in local goats of Southwestern China was elucidated by identifying associations between polymorphisms in *ASIP*, *AHCY*, and *ITCH* genes and diverse pigmentation patterns, providing tools for breed authentication and marker-assisted selection.

### 2.3. Growth, Carcass, and Meat Quality Traits

Growth performance, carcass composition, and intramuscular fat (IMF) deposition are economically critical polygenic traits, and multiple articles addressed their genetic and molecular basis through complementary approaches.

A GWAS study on Charolais cattle utilizing high-coverage whole-genome resequencing in a cohort of 240 animals identified 196 significant SNPs for growth traits and 29 for carcass traits, pinpointing *ACOX1* as a strong candidate gene influencing backfat thickness and body weight and demonstrating the resolution advantage of whole-genome sequencing compared with chip-based platforms [12]. In goats, missense mutations in the *COLQ* gene were found to affect neuromuscular junction stability with downstream consequences for carcass traits in Leizhou Black Goats. A histological and transcriptomic characterization of postnatal longissimus dorsi development in Ziwuling Black Goats mapped the transition from myofiber proliferation to hypertrophy, thereby providing a timeline for nutritional intervention windows. SNPs in the promoter region of the myogenin (*MyoG*) gene were shown to alter transcription factor binding sites and were significantly associated with body weight and length in Guizhou White Goats, illustrating how regulatory variants can exert quantitative effects on growth traits.

IMF deposition and fatty acid composition have received particular attention through multi-omics approaches. CIDEA was characterized as a key regulator of lipid deposition in goat intramuscular preadipocytes, promoting IMF accumulation via activation of the focal adhesion pathway while simultaneously suppressing cell proliferation. Transcriptome–metabolome integration in three-way crossbred sheep and in lambs identified coordinated regulation of lipid biosynthesis pathways; in lambs, the *MYH7* gene was found to drive a shift toward slow-twitch myofibers that enhances the accumulation of unsaturated fatty acids beneficial to meat nutritional quality. A study on Duroc pigs, included for its comparative insights into ruminant muscle biology, integrated bulk RNA sequencing and LC-MS proteomics across three postnatal muscle types and identified nine critical genes underlying muscle-type heterogeneity, with findings that informed parallel cattle and sheep research.

### 2.4. Reproductive Performance and Litter Size

Reproductive efficiency directly determines flock and herd profitability, and several articles expanded the catalog of functional genes and non-coding RNAs amenable to selection for prolificacy improvement.

The NR4A1 transcription factor was characterized in the Shaanbei White Cashmere Goat, where promoter polymorphisms affecting transcription factor binding were associated with litter size, implicating *NR4A1* in the regulatory network governing ovarian function. In Hetian sheep, the *ID2* gene was demonstrated to promote granulosa cell proliferation and modulate steroid hormone secretion, with specific SNPs directly linked to increased litter size, providing molecular targets for marker-assisted selection. Whole-genome sequencing of Dorper × Hu hybrid sheep, combined with selective sweep analysis and GWAS, identified candidate genomic regions harboring genes such as *SLC24A2* and *ABCA1* that were significantly associated with high litter size, establishing a genomic reference for improving prolificacy in this widely bred hybrid.

Non-coding RNA regulation of reproduction was addressed by two notable studies. In dairy goat endometrium, circRNA3890 was found to function as a molecular sponge for miR-26b-3p, de-repressing the MDM4/p53 signaling axis to establish endometrial receptivity—a prerequisite for successful embryo implantation with direct implications for embryo-transfer programs. Proteomic profiling of semen from Duolang sheep rams carrying different FecB genotypes (wild-type, heterozygous BB+, and homozygous BB) via DIA quantitative proteomics revealed that differentially expressed seminal proteins are enriched in innate immune response and oxidative phosphorylation pathways, providing insight into how a major prolificacy gene affects male reproductive biology and sperm quality.

### 2.5. Milk Production, Genomic Diversity, and Breeding Strategy

Milk production and mammary gland biology were addressed in a small but impactful cluster of studies. An integrated analysis of whole-genome sequencing data from 78 water buffaloes, deploying multiple GWAS models (cBLUP, GMATs, and BayesR), identified *MAPK10* as a candidate gene for 305-day milk yield, offering a molecular entry point for improving dairy buffalo productivity. An integrated transcriptomic study combining data from 77 *Bos taurus* and 77 *Ovis aries* mammary gland samples, analyzed through WGCNA and protein–protein interaction networks, identified *RPS15* and *RPL7A* as robust hub genes regulating species-specific differences in milk quality and lactation performance, underscoring the value of cross-species comparative transcriptomics.

Genetic diversity assessment was addressed in two complementary studies. ROH analysis of the Kazakh Fat-Tailed Coarse-Wool sheep characterized genomic inbreeding levels, identified selection signatures associated with muscle formation (*MYF5*) and fat metabolism (*PRDM16*), and provided population-level insights relevant to conservation and selective breeding [13]. In crossbred cattle populations of the Colombian Caribbean, SNP-based PCA and ROH analyses demonstrated that despite the absence of formal breeding programs, these populations retain high genetic diversity and carry favorable allele frequencies for both milk and meat production—findings with immediate practical implications for sustainable breeding in tropical environments [14].

Genomic selection methodology was advanced by studies comparing ABLUP, GBLUP, and single-step GBLUP (ssGBLUP) for early growth traits in Inner Mongolian cashmere goats, demonstrating that ssGBLUP—which seamlessly blends pedigree and genomic data—outperformed traditional approaches [15]. Extending these methodological advances, machine learning algorithms including Random Forest, Gradient Boosting Decision Tree, XGBoost, and LightGBM were benchmarked against GBLUP for cashmere fiber traits in the same breed; hyperparameter-tuned LightGBM captured non-linear marker interactions and achieved superior prediction accuracy for fiber diameter and cashmere yield [10,16]. Genetic parameter estimation and model optimization for early growth traits in Yunnan semi-fine wool sheep provided a methodological foundation for national-level breeding program optimization in this breed.

### 2.6. Genome Editing, and Multi-Omics for Complex Traits

A forward-looking cluster of contributions addressed the emerging frontiers of epigenomics, non-coding RNA biology, CRISPR-based genome editing, and spatial multi-omics, collectively pointing toward the next generation of breeding tools [17].

The *MSTN* gene-edited cattle study combined whole-genome bisulfite sequencing (WGBS) and transcriptomics to reveal that loss of *MSTN* not only induces the expected skeletal muscle hypertrophy [18] (with improved shear force and cooking loss metrics) but also unexpectedly reshapes the transcriptomic and DNA methylation landscapes of cardiac and smooth muscle, with axon guidance signaling emerging as a pleiotropically affected pathway. This study highlighted both the transformative potential and the need for systematic phenotypic monitoring when applying gene-editing technologies in livestock. In a parallel disease-resistance application, base editing was used to introduce a targeted mutation at the 2SP site of the bovine *NRAMP1* promoter; dual luciferase assays confirmed that this endogenous edit—achieved without synthetic selection markers—significantly enhanced *NRAMP1* expression upon Mycobacterium tuberculosis challenge while maintaining baseline levels in uninfected cells, offering a cleaner and safer route to tuberculosis-resistant cattle than transgenic approaches [8,9].

A comprehensive review on innovations in cattle breeding technology surveyed the current state of CRISPR-Cas applications in livestock, critically examining regulatory challenges and advocating for science-based policy frameworks to facilitate the translation of gene-editing discoveries into commercial breeding programs. For complex growth traits in cattle, a multi-omics study linking the blood transcriptome, fecal metabolome, and gut microbiome identified a cross-omics regulatory axis where host genes (*RADIL*, *ZBTB20*) interact with gut microbes (Oscillospira) to modulate bile acid metabolism and influence average daily gain, exemplifying how host–microbiome interactions can be integrated into genomic models [7]. Spatial multi-omics profiling of the Qianqiu goat gut—combining 16S rRNA sequencing, untargeted LC-MS metabolomics, and WGCNA—revealed a distinctive enrichment of methanogens in the small intestine that shapes host lipid metabolism in a region-specific manner. Metabolomic analysis of cauda epididymal fluid in yaks and cattle identified species-specific differences in antioxidant capacity and energy metabolites relevant to sperm maturation physiology in high-altitude environments.

## 3. Conclusions

The 35 articles assembled in this Topic collectively chart the frontiers of molecular genetics and breeding in cattle, sheep, and goats, and together they signal a clear paradigm shift: from the reductionist study of individual genes toward holistic, systems-biology approaches that integrate genomics, transcriptomics, epigenomics, proteomics, and metabolomics with advanced computational frameworks including machine learning and spatial multi-omics. GWAS, GS, and multi-omics integration are moving from proof-of-concept in major commercial breeds to practical implementation in locally adapted and indigenous populations, broadening the beneficiaries of genomic improvement.

Several cross-cutting themes deserve emphasis. First, non-coding RNA biology—particularly circRNA—has emerged as a productive research frontier in ruminant genetics, with roles demonstrated here in wool follicle development, endometrial receptivity, and beyond, confirming that the regulatory genome is as important a target as the protein-coding genome. Second, epigenomic approaches, exemplified by WGBS studies on *MSTN*-edited cattle and multi-omics analyses of growth traits, indicate that DNA methylation and chromatin state are tractable layers of biological information that should be incorporated into next-generation breeding models. Third, the application of CRISPR-Cas genome editing to both production traits (*MSTN*) and disease resistance (*NRAMP1*) demonstrates that animal scientists are no longer merely observing natural genetic variation but are becoming active architects of livestock genomes.

Looking ahead, four priorities stand out. First, scaling GS to smallholder systems in developing regions will require cost-effective low-density panels and robust imputation pipelines. Second, integrating microbiome and epigenomic data into breeding indices offers a promising avenue for capturing “missing heritability” and improving resilience to nutritional and environmental variation. Third, CRISPR-based functional validation of candidate genes identified through GWAS and multi-omics—particularly those affecting hypoxia tolerance, endometrial receptivity, and lipid metabolism—will accelerate the translation from discovery to commercial application. Fourth, ethical and regulatory frameworks for genome-edited livestock must evolve in parallel with technical advances to ensure societal acceptance and responsible deployment.

It is our hope that this collection will serve as both a valuable resource for researchers and a catalyst for continued innovation in the molecular genetics and precision breeding of ruminant livestock.
